# Association between the *FTO* SNP rs9939609 and Metabolic Syndrome in Chilean Children

**DOI:** 10.3390/nu13062014

**Published:** 2021-06-11

**Authors:** Rafael Molina-Luque, Natalia Ulloa, Manuel Romero-Saldaña, Martin Zilic, Andrea Gleisner, Fabián Lanuza, Guillermo Molina-Recio

**Affiliations:** 1 Grupo Asociado de Investigación Estilos de Vida, Innovación y Salud, Instituto Maimónides de Investigación Biomédica de Córdoba (IMIBIC), 14004 Córdoba, Spain; rafael.moluq@gmail.com (R.M.-L.); manuelromerosal@gmail.com (M.R.-S.); en1moreg@uco.es (G.M.-R.); 2 Departamento de Enfermería, Farmacología y Fisioterapia, Facultad de Medicina y Enfermería, Universidad de Córdoba, 14004 Córdoba, Spain; 3 Centro de Vida Saludable, Universidad de Concepción, Concepción 4070386, Chile; 4 Departamento de Bioquímica Clínica e Inmunología, Facultad de Farmacia, Universidad de Concepción, Concepción 4070386, Chile; 5 En Representación de el Consorcio ELHOC Chile (Epidemiology of Lifestyle and Health Outcomes in Chile), Concepción 4070386, Chile; f.lanuza89@ub.edu; 6 Facultad de Medicina, Universidad de Concepción, Concepción 4070386, Chile; mzilic@udec.cl; 7 Departamento de Pediatría, Facultad de Medicina, Universidad de Concepción, Concepción 4070386, Chile; andrea.gleisner@gmail.com; 8 Biomarkers and Nutrimetabolomics Laboratory, Department of Nutrition, Food Sciences and Gastronomy, Nutrition and Food Safety Research Institute (INSA), Faculty of Pharmacy and Food Sciences, University of Barcelona, 08028 Barcelona, Spain; 9 Centro de Epidemiología Cardiovascular y Nutricional (EPICYN), Facultad de Medicina, Universidad de La Frontera, Temuco 4781218, Chile

**Keywords:** *FTO* gene, metabolic syndrome, children

## Abstract

Background: The increasing prevalence of obesity in children has raised the incidence of Metabolic Syndrome (MetS) in this age group. Given the short- and long-term health impact of MetS, it is essential to prevent its onset by detecting its main triggers. Besides, genetic factors play an essential role in influencing which individuals within a population are most likely to develop obesity in response to a particular environment. In this regard, a common variation in the FTO gene is reproducibly associated with BMI and obesity from childhood and the genetic load has been linked to several cardiovascular risk factors, highlighting the FTO single nucleotide polymorphism (SNP) rs9939609. Therefore, this study aimed to establish the relationship between the FTO SNP rs9939609 and MetS. Methods: A cross-sectional study was carried out on 220 children from the Biobío region (Chile). MetS diagnosis was established through the modified Cook criteria, using prevalence ratios, COR curves, and linear regressions to determine its association with MetS and its components. Results: The prevalence of MetS was significantly increased among carriers of the risk allele (A): TT, 20.2%; TA, 25.4%; AA, 44.7% (*p* = 0.006). Also, the presence of A was associated with altered MetS-related variables. Conclusions: The FTO SNP rs9939609 was associated with a raised prevalence of MetS among A allele carriers, and was higher in the homozygous genotype (AA).

## 1. Introduction

Obesity is a highly prevalent chronic health problem associated with an increased risk of type 2 diabetes mellitus, heart disease, metabolic syndrome, hypertension, stroke, and certain forms of cancer. This problem affects over 340 million children and adolescents. The prevalence of obesity has increased approximately from 4% to 18% in the last 40 years worldwide [1]. Furthermore, it is estimated that the number of obese individuals will reach 1.12 billion or 20% of the world’s adult population in 2030 [2,3].

The childhood obesity period represents a priority public health problem associated with developing chronic metabolic diseases [4,5]. The prevalence increased in both developed and developing countries [6]. In Chile, childhood obesity has increased steadily since the late 1980s [7,8]. In 2019, the National Board of School Aid and Scholarships of the Government of Chile (JUNAEB) reported that the prevalence of overweight and obesity at different school levels ranges from 47.6% to 60% [9]. In the development of obesity, it is essential to keep in mind modifiable and non-modifiable risk factors [10]. Non-modifiable factors include sex, age, ethnicity, Tanner stage, and genetic polymorphisms [11,12]. Indeed, Frayling et al. showed that the rs9939609 polymorphism of the FTO gene (Fat Mass Obesity-associated gene) was associated with the body mass index (BMI) [13]. After this research, more than 97 genetic polymorphisms associated with a higher BMI had been reported [14], and a meta-analysis summarized the existence of 738 SNPs associated with various adiposity markers [15]. Despite the large number of genetic variants related to obesity, the rs9939609 polymorphism of the FTO gene has been more studied due to its effect on increasing BMI. This association has been confirmed in different populations of adults and children worldwide [16,17,18,19].

The prevalence of metabolic syndrome (MetS) in childhood is increasing; likewise there is a rise in obesity and metabolic diseases [19,20]. According to the Joint Task Force statement, MetS has been diagnosed with three of these five risk factors: abdominal obesity, high blood pressure, hypertriglyceridaemia, low HDL, and fasting hyperglycemia [21]. Currently, there are no consensus guidelines for metabolic syndrome in the pediatric population [22,23]. Concerning the association of metabolic syndrome with polymorphisms of the FTO gene, a meta-analysis concluded that the FTO gene might play a critical role in leading to MetS [24].

Previously, our group reported that the rs9939609 variant of the FTO gene was associated with a higher risk of increased BMI in Chilean children [19], and recently we reported the association of this same variant of the FTO gene with increased general and central adiposity [25]. However, we did not analyze whether the risk of metabolic syndrome is modified with this variant of the FTO gene in this sample. Consequently, the objective of this study was to investigate the association between the rs9939609 in FTO gene and metabolic syndrome and to evaluate the components of metabolic syndrome in this same Chilean child population.

## 2. Materials and Methods 

### 2.1. Design, Population, Sample

We used data from a cross-sectional study in children sample from an urban area in the Biobío Region of Chile. For an expected MetS prevalence of 22.7% [26], an accuracy of 6%, and a 95% confidence interval, the calculated minimum sample size was 188. Finally, 220 children were studied. Children from an age range of 6 to 11 years were included. Children suffering from any chronic pathology and those whose parents or legal guardian did not sign the informed consent were excluded.

### 2.2. Study Variables and Measurements

MetS was diagnosed following the criteria suggested by Cook [27]. These define MetS by the appearance of three or more of the following alterations: waist circumference (WC) ≥ 90th percentile, systolic blood pressure (SBP) or diastolic (DBP) ≥ 90th percentile, high-density lipoprotein cholesterol (HDL-C) ≤ 40 mg/dL, triglycerides (TG) ≥ 110 mg/dL, but at fasting blood glucose levels ≥ 100 mg /dL, the value defined by the American Diabetes Association was considered [28]. 

In addition, a set of independent variables were collected: age (years), sex (woman/man), weight (kg), height (cm), BMI (kg/m^2^), waist–height index (WHtR), fat-free mass (FFM, kg), fat mass (FM, kg), body fat percentage (BF%), systolic (SBP, mmHg) and diastolic blood pressure (DBP, mmHg), glucose (mg/dL), high-density lipoproteins cholesterol (HDL-C, mg/dL), low-density lipoproteins cholesterol (LDL-C, mg/dL), TG (mg/dL), and total cholesterol (mg/dL).

Anthropometric were measured following the criteria of the standardized anthropometric manual [29]. Height was assessed without shoes, using 0.1 cm precision wall-mounted stadiometers (Seca, model 208). Bodyweight was measured with light clothing and without shoes on a Tanita scale (TANITA TBF-300, TANITA, Tokyo, Japan) with an accuracy of 1 g. The WC was taken at the midpoint between the last rib and the upper border of the iliac crest with a non-elastic flexible tape (Seca, model 201) with an accuracy of 0.1 cm. Body composition was evaluated by bioelectrical impedance analysis (TANITA TBF-300, TANITA, Tokyo, Japan). Blood pressure was taken following established recommendations [30], using a calibrated digital sphygmomanometer (OMRON M3, OMRON, Kyoto, Japan). Blood pressure was measured three times, on the right arm, with the child seated, with an interval of 5 min between each measurement and using the mean of the last two measures. Trained nutritionists took all measurements. Each measurement was repeated three times, and the mean was registered. According to the Tanner criteria, the pubertal stage was established by a pediatrician [31].

BMI was calculated as body weight divided by height in meters squared. The BMI z score, based on age and sex, was calculated according to the WHO definitions. The children were classified following the WHO criteria as normal weight: Z-Score BMI> − 2DE and < + 1SD; overweight: Z-Score IMC> + 1SD or < + 2SD; Obesity: Z-Score BMI> + 2SD [32]. Determinations of abdominal obesity and arterial hypertension were made using the reference tables according to age and sex, considering height in blood pressure [33,34].

### 2.3. Plasma Determinations

We collected 4 mL of fasting venous blood after an overnight fast, between 8–12 h of fasting. We used colourimetric methods to measure total cholesterol, HDL-C, LDL-C, TG, and glucose levels with analysis kits (Cobas C111 Roche, Indianapolis, IN, USA). Plasma insulin was measured using a commercial ELISA kit (Linco Research, St. Charles, MO, USA) using a multi-reader, Synergy 2 (Biotek, Winooski, VT, USA).

### 2.4. Determination of Allelic Variants of the FTO Gene

The single nucleotide polymorphism (SNP) (rs9939609) of the FTO gene was determined using genomic DNA extracted from leukocytes, using the Mini Kit QIAamp DNA Blood (Qiagen GmbH, Hilden, Germany), according to the manufacturer’s protocol. Polymerase chain reaction (PCR) amplifications were performed on a real-time PCR thermocycler, Rotor-Gene 6500 (Corbett Research, Sydney, Australia). For this, previously described primers were used (direct: 5′d AACTG GCTCTTGAATGAAATAGGATTCAGA 3′ and inverse: 5′d AGAGTAACAGAGACTATCCAAGTGCATCAC 3′) [35], under a standardized protocol [18]. Genotype identification was performed by comparison (confidence intervals, 95% CI) of the fusion data with standard genotypes identified by sequencing analysis in the Department of Ecology, Faculty of Biological Sciences at Pontificia Universidad Católica de Chile. To confirm the existence of a single PCR product, 3% agarose gel electrophoresis was performed. All analyses of the samples were performed in duplicates, with 98% success in determining the genotype. 

### 2.5. Ethical and Legal Aspects

We conducted the study following the Declaration of Helsinki (1964), in the Convention of the Council of Europe relating to human rights and biomedicine (1997), in the Universal Declaration on the human genome and human rights (UNESCO, 1997), as well as fulfilling the requirements established in the Chilean legislation in the field of biomedical research, the protection of personal data and bioethics, according to Decree No. 114 of 2010, which approves the regulation of the Law No. 20,120, and which was modified and updated in Decree 30 January of 14 January 2013. The Bioethics Committee of the Vice-Rectory of Research of the University of Concepción approved the study protocol (352-2019).

### 2.6. Statistical Analyses

The continuous variables were presented as mean and standard deviation (SD), and categorical variables as absolute frequency and percentages. The goodness of fit of continuous variables to a normal distribution was tested using the Kolmogorov–Smirnov test with Lilliefors’ correction. For comparing more than two means, the analysis of variance (ANOVA) with Bonferroni for the post hoc comparison was performed. In case the data did not fit a normal distribution, the non-parametric Kruskal–Wallis version was computed. For categorical variables, the Chi-square test (2) was used. Prevalence ratios were calculated. The linear trend test was performed to determine significant differences in the outcome across the FTO genotype (the TT genotype was coded as 0 and used as the reference group, TA was coded as 1, and the AA was coded as 2).

The association between the FTO genotype and continuous outcomes included in or directly or indirectly related to MetS (Weight, WC, WHtR, BMI, FM, BF%, Insulin, HOMA-IR, TG, and HDL) was estimated through multiple linear regressions. Also, standard error, adjusted coefficient of determination, F statistic, linearity analysis, and residues were analyzed to determine the goodness of fit.

For all statistical analyses, an alpha error probability of less than 5% (*p* < 0.05) was accepted and the confidence interval was calculated at 95%. The software programs IBM SPSS Statistics 25.0 (IBM, Chicago, IL, USA), EPIDAT 4.2 (Department of Sanidade, Xunta de Galicia, Galicia, Spain) were used for the statistical analysis.

## 3. Results

### 3.1. Sample Description

The cohort characteristic is presented in Table 1. In summary, of the 220 children included in the study, 50% was females. The mean age was 9.1 (SD 1.2) years. The prevalence of obesity was 33.3%, whereas the prevalence of central obesity based on WHtR was 57.7%. The average BMI (z-score) was 1.52 (SD 0.83), and the body fat percentage was 30.0% (SD 9.4). The fasting glucose and insulin concentrations were 88.3 mg/dL (SD 8.56) and 8.6 μU/mL (SD 6.85), respectively. For lipids profile, the average total cholesterol concentration was 181.84 mg/dL (SD 33.1) and 108.26 mg/dL (SD 28.9) for LDL-C. For TG, the average concentration was 123.1 mg/dL (SD 73.87). Based on the Cook criteria for MetS, the prevalence of high blood pressure was 28.5% (95%CI, 22.654–34.947), abdominal obesity (WC > 90th percentile) was 59.1% (95%CI, 52.280–65.653), higher TG (TG > 110 mg/dL) was 47.7% (95%IC, 40.970–54.547), low HDL-C (HDL < 40 mg/dL) was 20% (95%IC, 14.924–25.903), and high glycemia (glucose > 100 m/dL) was 9.5% (95%IC, 5.979–14.158). Finally, the prevalence of metabolic syndrome was 26.8% (95%CI, 21.085–33.187).

### 3.2. FTO Genotype and Metabolic Syndrome

Furthermore, the presence of the AA variant of the FTO gene was related to a worse anthropometric and cardiometabolic condition. The children who presented it showed a higher average weight (*p* = 0.004), WC (*p* = 0.015), WHtR (*p* = 0.041), BMI (*z*-score) (*p* = 0.001) and BF% (*p* = 0.004). Concerning to metabolic state, those with two risk alleles of rs9939609 polymorphism had higher mean values of fasting insulin (*p* < 0.001), HOMA-IR (*p* = 0.004) and TC (*p* = 0.051). Also, this group showed significantly lower means of HDL-C (*p* = 0.001). Finally, this situation turned into a higher prevalence of MetS in this group (*p* = 0.006), with 44.7%, compared to 23% and 15% present in children with TT (Wild type) and TA (Heterozygous) genotypes, respectively. A more detailed description of these results can be observed in Table 2.

The presence of MetS components seems to be related to the type of alleles present in the rs9939609 polymorphism of the FTO gene of the participants (Table 2). Significantly different prevalence ratios were obtained for the HBP component (*p* < 0.001), WC (*p* = 0.006), fasting glucose (*p* = 0.004), and HDL-C (*p* < 0.001). Besides, significant linear trends were found in the prevalence ratios (Level 1: TT (reference), Level 2: TA and Level 3: AA), with WC (*p* = 0.001) increasing and the presence of the HDL-C component decreasing (*p* = 0.048) (Table 3).

Significant linear trends have also been found between the number of components and the genotype of the FTO gene. This trend is decreasing for children with none (*p* = 0.046) and increasing for those showing three or four components (*p* = 0.045 and *p* = 0.048, respectively). As a result, we found that this linear trend is also observed in the diagnosis of MetS, which is 2.21 (*p* = 0.006) higher in children with genotype AA (Table 4).

Furthermore, according to Tanner stage, adjusted multiple linear regression analysis by age, gender, and physical development showed that the presence of the risk allele of rs9939609 of the FTO gene significantly increases the probability of suffering from metabolic syndrome through its influence on its different components. In the case of AA genotype, results have shown a clear association with increased WC (β = 6.934; *p* < 0.001), TG (β = 42.629; *p* < 0.001), insulin resistance estimated through HOMA-IR (β = 1.010; *p* < 0.001) and a decrease in HDL-C levels (β = –6.912; *p* < 0.001). Harmful effects on the nutritional status of children have also been detected, with a significant increase in weight (β = 7.289; *p* < 0.001), BMI (β = 2.934; *p* < 0.001) and body fat percentage (β = 6.178; *p* < 0.001) of subjects with this genotype. Finally, children with the TA genotype were also at higher risk than those with the TT genotype for increased weight, WC, WHtR, BMI, BF%, and insulin resistance (Table 5).

**Figure 1:**
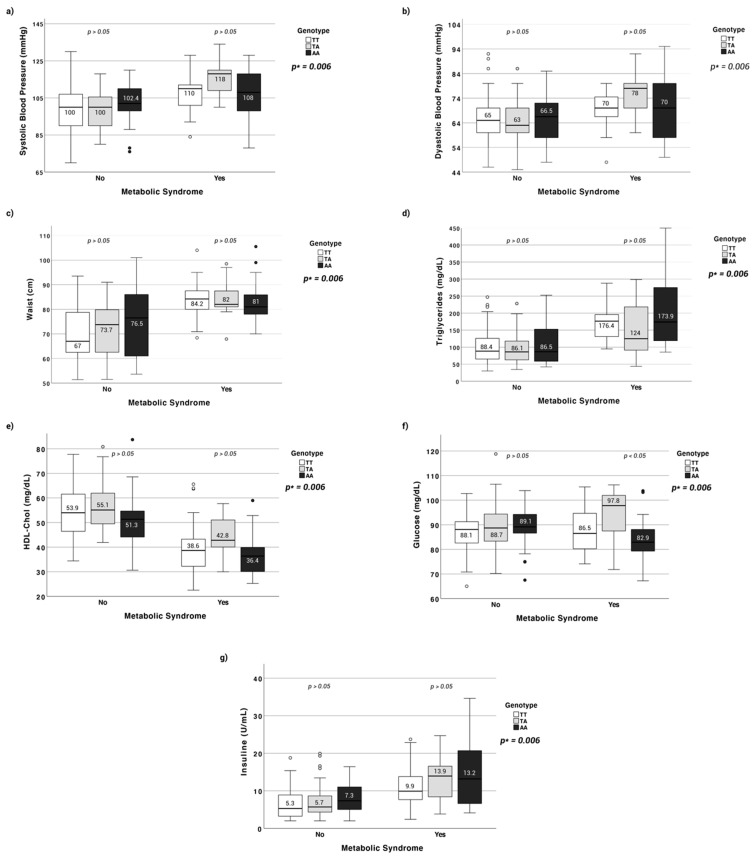
(**a**) Systolic Blood Pressure average according to Metabolic Syndrome and Genotype (**b**) Diastolic Blood Pressure average according to Metabolic Syndrome and Genotype (**c**) Waist Blood Pressure average according to Metabolic Syndrome and Genotype (**d**) Triglycerides average according to Metabolic Syndrome and Genotype (**e**) HDL-Cholesterol average according to Metabolic Syndrome and Genotype (**f**) Glucose average according to Metabolic Syndrome and Genotype (**g**) Insulin average according to Metabolic Syndrome and Genotype. The upper limit of each box corresponds to the third quartile value (75% of the data) and the lower limit to the first quartile (25%). The line into the box represents the median. Two vertical bars (whiskers) are included. The length of the vertical bars (whiskers) is determined by these values: Q1 − [1.5 × the interquartile range] for the lower one and Q3 + [1.5 × the interquartile range] for the upper one. Symbols represent outliers and extreme values (distances exceeding those defined for whiskers). (*p*): indicates the *p*-value of the difference in means (ANOVA or Kruskal-Wallis) between the different genotypes and for each group according to whether or not metabolic syndrome is present. (*p* *): indicates the *p*-value of the association between the different genotypes and the presence or absence of metabolic syndrome.

The relationship between the different components of the metabolic syndrome, the presence or absence of this syndrome, and the FTO gene variant is shown in Figure 1. The means of TG are higher in children with MetS and with genotype AA, while those of HDL-C are lower in this group. Overall, glucose is lower in participants with MetS and even more so in those with the genotype AA. However, in this group, insulin levels are much higher, suggesting the activation of a compensation system that seems to be effective in this studied group.

## 4. Discussion

This research aimed to determine whether the different genotypes associated with the polymorphism rs9939609 correlate to an increased susceptibility to MetS in children. 

Previous reports have shown the relationship between the A allele rs9939609 of the FTO gene and the increased risk of overweight and obesity in children, regardless of ethnicity, gender, or developmental stage [19,25,36]. This risk, which is higher for homozygous (AA), is demonstrated in both general and central adiposity parameters [25]. In other words, the presence of allele A is associated with a more significant deposit of fat, which is related to a higher number of cardio-metabolic complications [37]. This fact is explained by the increase in adipose tissue, which is associated with higher insulin resistance (IR) and represents the primary physiopathological mechanism of MetS [38]. Our results have shown that IR increases significantly between the different genotypes, being higher in AA (β = 1.010, *p* < 0.001) [25]. This trend leads to high insulinemia as a compensatory mechanism that prevents hyperglycemia in children. For this reason, high basal blood glucose is the least prevalent component of MetS (9.5%). Despite this, this criterion was more present among carriers of the genotype TA and AA (*p* = 0.004).

Although the increase of insulinemia limits the development of hyperglycemia, IR is involved in the rest of the MetS-linked disorders [39]. Several authors have shown that IR affects lipid metabolism, altering HDL-C levels [40,41]. Furthermore, it is involved in the genesis of hypertension [42]. These alterations are more frequent in our sample than hyperglycemia, with 20% and 28.5%, respectively. In terms of distribution among genotypes, all MetS criteria were significantly more prevalent in AA, except in hyper-TG. Each copy of the A allele represented a significant linear increase in the prevalence ratios of abdominal obesity criteria and HDL-C alteration. This influence of the genotype on the variables involved in MetS was also observed in the adjusted multivariate analysis. 

The proportion of children with 0–4 components was not significantly different between the different genotypes. However, the prevalence ratios of those with three and four components increased significantly with each copy of the A allele. This situation resulted in the prevalence of MetS being distributed differently between the different genotypes (*p* = 0.004), growing linearly in the TA (1.26 95% CI 0.71–2.23) and the AA (2.21 (95%CI 1.36–3.59), and taking TT as reference (*p* = 0.002). These results are similar to those found by Kelishadi et al. in their meta-analysis, in which they stated the relationship between the FTO gene and MetS [43]. Other authors have concluded that this association is not present in the pediatric population but in the adults [44]. However, considering that the genotype does not change throughout life, it can be thought that these results are influenced by the low methodological quality of the studies evaluated. Finally, other SNP interactions associated with MetS and its components, such as WC, should also be considered because they could increase the risk of MetS [45,46].

Although the association found between the rs9939609 SNP and MetS is in line with evidence from other authors, and remains constant in different ethnicities [47,48,49,50,51,52,53], the mechanism by which the polymorphism influences the development of MetS and the alteration of its components is unclear [47,48,54]. One hypothesis is that its role in lipogenesis, widely described [55,56], derives from the associated metabolic alterations, but this has not been demonstrated [47,57]. In this regard, our results show that for each risk allele (A), there is a significant increase in the amount of fatty tissue and abdominal obesity measured by WC. Its relation with the impairment of lipid metabolism, especially in TG and HDL-C, has also been suggested [48,49], which has been observed in our results. On the other hand, it has been evidenced that being a carrier of the risk allele of the rs9939609 SNP is associated with an alteration of glucose metabolism [54,58], which could be a precursor of changes in the regular brain control of adiposity and, therefore, derive in MetS [59]. However, one aspect on which most authors agree is that further studies are needed to identify the mechanism that defines this association [47,52].

However, although the underlying mechanism has not yet been clarified, the relationship between this polymorphism and MetS seems clear. Hence authors consider that it is essential to use this knowledge for preventing the development of MetS. Although genetic load increases the risk of developing MetS, it has been shown that lifestyle factors, such as feeding and physical activity, can modulate its expression. Concerning diet, high saturated fatty acid (SFA) intake and a low ratio between polyunsaturated fatty acid (PUFA) and SFA intake have been found to increase the risk of obesity, especially in patients with MetS [60]. Furthermore, substituting SFA for PUFA in patients carrying the risk allele (A) decreases the risk of cardiovascular disease [61]. Controlling fat intake would modulate the rs9939609 polymorphism and could also help to reduce the effect of this macronutrient on other genetic variants related to metabolic alterations, such as the rs5082 polymorphism of the APOA2 gene and its influence on insulin resistance [62]. In addition, it has recently been found that adapting the intake of different macronutrients to the other genotypes linked to several FTO gene polymorphisms (rs9939609, among them) modifies the risk of obesity and metabolic disorders [63]. This fact means that carriers of the risk allele of the rs9939609 polymorphism, or other FTO gene polymorphisms, could be helped through individualized nutritional interventions [63,64].

On the other hand, physical activity is another lifestyle component that should be taken into account to improve patients’ health. It has been observed that the association between the rs9939609 SNP and MetS is higher in subjects with a low level of physical activity [65]. However, evidence shows that performing physical activity can significantly attenuate the rs9939609 SNP of the FTO gene [66,67,68] and other genes associated with obesity and metabolic alterations [69].

## 5. Limitations

A larger sample size would reinforce the findings, clarifying some results that might be affected by a limited number of cases. In addition, no variables related to the dietary pattern of the subjects were collected, so the effect of the diet on the participants could not be evaluated. Future research should develop prospective methodologies to determine the risk of MetS based on the genotype determined by the rs9939609 SNP. The literature on this topic is not recent, which makes it difficult to compare to our results.

## 6. Conclusions

In summary, we confirm the association of rs9939609 polymorphism of the FTO gene the presence of MetS. Specifically, for each copy of the A allele, an increase in the susceptibility to develop MetS has been observed. These results reinforce the idea that the polymorphism rs9939609 of the FTO gene could play a role in the genesis of MetS from childhood and could be considered in the context of personalized prevention.

## Figures and Tables

**Table 1:** Descriptive characteristics of the sample and according to the FTO genotype.

	Mean or *n* (SD or %)	95% Confidence Interval
Sex		
Male	110 (50)	43.205–56.795
Female	110 (50)	43.205–56.795
Age (years)	9.09 (1.27)	8.921–9.259
Tanner		
Prepubertal	156 (70.6)	64.106–76.509
Pubertal	65 (29.4)	23.491–35.894
Weight (kg)	41.18 (10.82)	39.742–39.742
Height (cm)	135.18 (9.03)	133.98–136.38
WC (cm)	74.55 (11.56)	73.014–76.086
WHtR	0.55 (0.07)	0.541–0.559
WHtR ≥ 0.55		
Yes	127 (57.7)	50.905–64.338
No	93 (42.3)	35.662–49.095
BMI	1.52 (0.83)	1.410–1.630
Obesity		
Yes	73 (33.2)	26.997–39.827
No	147 (66.8)	60.173–73.003
BF%	30.01 (9.46)	28.753–28.753
FM (kg)	13.34 (6.75)	12.443–14.237
FFM (kg)	28.38 (5.07)	27.706–29.054
SBP (mmHg)	101.55 (12)	99.955–103. 145
DBP (mmHg)	66.07 (10.65)	64.655–67.485
HBP		
Yes	63 (28.5)	22.654–34.947
No	158 (71.5)	65.053–77.346
Glucose (mg/dL)	88.28 (8.56)	87.143–89.417
Insulin (μU/mL)	8.6 (6.85)	7.690–9.510
HOMA-IR	1.86 (1.36)	1.679–2.041
TG (mg/dL)	123.1 (73.87)	113.285–132.915
TC (mg/dL)	181.84 (33.1)	177.442–186.238
HDL-C (mg/dL)	50.51 (11.66)	48.961–52.059
LDL-C (mg/dL)	108.26 (28.9)	104.420–112.100
MetS-Comp		
0	51 (23.2)	17.774–29.327
1	53 (24.1)	18.597–30.297
2	57 (25.9)	20.253–32.227
3	40 (18.2)	32.227–23.925
4	19 (8.6)	5.280–13.158
MetS		
Yes	59 (26.8)	21.085–33.187
No	161 (73.2)	66.813–78.915

Tanner: physical development according to Tanner stage; WC: Waist circumference; WHtR: Waist-to-Height Ratio; BMI: Body Mass Index (z-score); BF%: Body Fat Percentage; FFM: Fat Free Mass; SBP: Systolic Blood Pressure; DBP: Diastolic Blood Pressure; HBP: High Blood Pressure (according to Cook); HOMA-IR: Homeostatic Model Assessment of Insulin Resistance; TG: Triglycerides; TC: Total Cholesterol; HDL-C: High Density Lipoprotein Cholesterol; LDL-C: Low Density Lipoprotein Cholesterol; MetS-Comp: Number of Metabolic Syndrome components showed (according to modified criteria from Cook); MetS: Presence of Metabolic Syndrome (according to modified criteria from Cook).

**Table 2:** FTO genotype and metabolic syndrome ^Δ.^

	TT	TA	AA	*p*
	Mean or *n* (SD or %)	Mean or *n* (SD or %)	Mean or *n* (SD or %)	
Sex				
Male	50 (43.9)	33 (55.9)	27 (57.4)	0.166
Female	64 (56.1)	26 (44.1)	20 (42.6)	
Age (years)	9.24 (1.18)	8.84 (1.33)	9.05 (1.37)	0.15
Tanner				
Prepubertal	79 (68.7)	44 (74.6)	33 (70.2)	0.721
Pubertal	36 (31.3)	15 (25.4)	14 (29.8)	
Weight (kg)	39.49 (10.15) *	40.89 (10.13)	45.64 (12.12) *	0.004
Height (cm)	135.04 (8.32)	134.15 (10.04)	136.82 (9.32)	0.31
WC (cm)	72.83 (11.11)	74.64 (11.2)	78.59 (12.29)	0.015
WHtR	0.54 (0.07) *	0.56 (0.07)	0.57 (0.07) *	0.013
WHtR ≥ 0.55				
Yes	57 (50)	37 (62.7)	33 (70.2)	0.041
No	57 (50)	22 (37.3)	14 (29.8)	
BMI	1.33 (0.82) *	1.62 (0.81)	1.83 (0.75) *	0.001
Obesity				
Yes	66 (57.9)	42 (71.2)	8 (17)	0.006
No	48 (42.1)	17 (28.8)	39 (83)	
BF%	28.3 (8.95) *	30.7 (8.89)	33.76 (10.36) *	0.004
FM (kg)	12 (6.12) *	13.42 (6.11)	16.54 (7.95) *	0.001
FFM (kg)	27.82 (4.79)	28.36 (5.01)	29.78 (5.63)	0.093
SBP (mmHg)	99.74 (11.78)	103.17 (11.5)	103.94 (12.62)	0.063
DBP (mmHg)	64.97 (10.16)	67.46 (10.17)	67 (12.23)	0.278
HBP				
Yes	24 (20.9)	23 (39)	16 (34)	0.028
No	91 (79.1)	36 (61)	31 (66)	
Glucose (mg/dL)	87.53 (7.66)	90.8 (9.65)	86.91 (8.68)	0.108
Insulin (μU/mL)	7.25 (4.52) *	8.51 (5.35) ^$^	11.95 (11.01) * ^$^	<0.001
HOMA-IR	1.57 (0.99)	1.94 (1.29)	2.46 (1.92)	0.004 ^Ç^
TG (mg/dL)	115.38 (56.01)	113.61 (77.09)	153.59 (97.8)	0.051 ^Ç^
TC (mg/dL)	184 (32.83)	181.5 (32.79)	177 (34.46)	0.476
HDL-C (mg/dL)	51.34 (11.79) *	53.11 (9.98) ^$^	45.27 (11.91) * ^$^	0.001
LDL-C (mg/dL)	110.46 (30.11)	107.94 (26.23)	103.33 (28.89)	0.362
MetS-Comp				
0	33 (28.9)	10 (16.9)	8 (17)	0.062
1	29 (25.4)	18 (30.5)	6 (12.8)	
2	29 (25.4)	16 (27.1)	12 (25.5)	
3	16 (14)	11 (18.6)	13 (27.7)	
4	7 (6.1)	4 (6.8)	8 (17)	
MetS				
Yes	23 (20.2)	15 (25.4)	21 (44.7)	0.006
No	91 (79.8)	44 (74.6)	26 (55.3)	

**^Δ^** Qualitative variables have been represented by frequency and percentage (in brackets). Quantitative variables have been represented by the mean and standard deviation (in brackets) Tanner: physical development according to Tanner stage; WC: Waist circumference; WHtR: Waist-to-Height Ratio; BMI: Body Mass Index (z-score); BF%: Body Fat Percentage; FFM: Fat Free Mass; SBP: Systolic Blood Pressure; DBP: Diastolic Blood Pressure; HBP: High Blood Pressure (according to Cook); HOMA-IR: Homeostatic Model Assessment of Insulin Resistance; TG: Triglycerides; TC: Total Cholesterol; HDL-C: High Density Lipoprotein Cholesterol; LDL-C: Low Density Lipoprotein Cholesterol; MetS-Comp: Number of Metabolic Syndrome components showed (according to modified criteria from Cook); MetS: Presence of Metabolic Syndrome (according to modified criteria from Cook) * ^$^ Significant differences between groups (Bonferroni post hoc) ^Ç^ Result of the Kruskal-Wallis test, applied when significant differences between variances. In these cases, post hoc tests have not been computed.

**Table 3:** Prevalence ratios of the components according to variability of the FTO gene ^Δ^.

Variable	TT	TA	AA	p ^*^	p ^**^
HBP	1 (Ref.)	0.53 (0.38–0.75)	1.6 (1.3–1.9)	<0.001	NS
TG	1 (Ref.)	0.82 (0.57–1.19)	1.26 (0.93–1.7)	NS	NS
WC	1 (Ref.)	1.25 (0.96–1.64)	1.53 (1.2–1.95)	0.006	0.001
Glucose	1 (Ref.)	3.9 (1.54–9.86)	1.22 (0.32–4.69)	0.004	NS
HDL-C	1 (Ref.)	0.35 (0.13–0.97)	1.98 (1.18–3,34)	<0.001	0.048

**^Δ^** Following Cook criteria to consider the presence of metabolic syndrome component: HBP (SBP or DBP > 90th percentile); TG >110 mg/dL; WC > 90th percentile; Glucose > 100 mg/dL and HDL-C < 40 mg/dL. HBP: High Blood Pressure; TG: Triglycerides, WC: Waist circumference; HDL-C: High Density Lipoprotein Cholesterol; NS: Non-significative. *p* * Significance of the differences inter-levels. *p* ** Significance Level of linear trend test.

**Table 4:** Prevalence ratios of the number of the Metabolic Syndrome components according to variability of the FTO gene ^Δ.^

Variable	TT	TA	AA	p ^*^	p ^**^
0	1 (Ref.)	0.58 (0.31–1.1)	0.59 (0.29–1.18)	NS	NS
1	1 (Ref.)	1.2 (0.73–1.97)	0.5 (0.22–1.13)	NS	NS
2	1 (Ref.)	1.07 (0.63–1.8)	1 (0.56–1.79)	NS	NS
3	1 (Ref.)	1.33 (0.66–2.68)	1.97 (1.03–3.77)	NS	0.045
4	1 (Ref.)	1.1 (0.34–3.62)	2.77 (1.06–7.21)	NS	0.042
5^ç^	1 (Ref.)	–	–	–	–
MetS	1 (Ref.)	1.26 (0.71–2.23)	2.21 (1.36–3.59)	0.006	0.002

**^Δ^** Following Cook criteria to consider the presence of metabolic syndrome component: BPH (SBP or DBP > 90th percentile); TG >110 mg/dL; WC > 90th percentile; Glucose > 100 mg/dL and HDL-C < 40 mg/dL. S: Non-significative. *p* * Significance of the differences inter-levels. *p* ** Significance Level of linear trend test. No-one in the sample showed all five components.

**Table 5:** Lineal multiple regression for assessing the relation between FTO types and the cardiometabolic status ^Δ^.

	β	Standardized β	*t*	*R*^2^ Adjusted	*p*
**Weight (kg)**					
TT	Reference				
TA	3.498	0.144	2.488	0.378	0.014
AA	7.289	0.277	4.828		<0.001
**WC (cm)**					
TT	Reference				
TA	3.711	0.142	2.212	0.207	0.028
AA	6.934	0.246	3.850		<0.001
**WHtR**					
TT	Reference				
TA	0.023	0.146	2.069	0.050	0.04
AA	0.039	0.228	3.25		0.001
**BMI**					
TT	Reference				
TA	1.584	0.168	2.488	0.124	0.014
AA	2.934	0.289	4.292		<0.001
**FM (kg)**					
TT			Reference		
TA	2.290	0.149	2.244	0.181	0.026
AA	5.100	0.310	4.693		<0.001
**BF%**					
TT	Reference				
TA	3.377	0.157	2.287	0.126	0.023
AA	6.178	0.268	3.931		<0.001
**Insulin (μU/mL)**					
TT	Reference				
TA	2.027	0.131	1.950	0.133	NS
AA	5.367	0.322	4.809		<0.001
**HOMA-IR**					
TT	Reference				
TA	0.516	0.169	2.494	0.124	0.013
AA	1.010	0.308	4.580		<0.001
**TG (mg/dL)**					
TT	Reference				
TA	3.777	0.023	0.324	0.062	NS
AA	42.629	0.237	3.406		0.001
**HDL (mg/dL)**					
TT	Reference				
TA	0.703	0.027	0.388	0.093	NS
AA	−6.912	−0.243	−3.556		<0.001

**^Δ^** They have only been calculated for the variables that showed significant differences in means during the bivariate analysis. Models Adjusted by age, sex and physical development according to Tanner stage. WC: Waist circumference; WHtR: Waist-to-Height Ratio; BMI: Body Mass Index; BF%: Body Fat Percentage; HOMA-IR: Homeostatic Model Assessment of Insulin Resistance; TG: Triglycerides; HDL-C: High Density Lipoprotein Cholesterol; NS: Non-significative.

## Data Availability

The data presented in this study are available on request from the corresponding author.
